# The impact of COPD on polyneuropathy: results from the German COPD cohort COSYCONET

**DOI:** 10.1186/s12931-020-1293-6

**Published:** 2020-01-20

**Authors:** K. Kahnert, M. Föhrenbach, T. Lucke, P. Alter, F. T. Trudzinski, R. Bals, J. I. Lutter, H. Timmermann, S. Söhler, S. Förderreuther, D. Nowak, H. Watz, B. Waschki, J. Behr, T. Welte, C. F. Vogelmeier, R. A. Jörres

**Affiliations:** 1 Department of Internal Medicine V – Pulmonology, University Hospital, LMU Munich, Comprehensive Pneumology Center Munich (CPC-M, member of German Center for Lung Research (DZL), Marchioninisr. 15, 81377 München, and Ziemssenstr. 1, 80336 Munich, Germany; 2 Institute and Clinic for Occupational, Social and Environmental Medicine, Comprehensive Pneumology Center Munich (CPC-M, member of German Center for Lung Research (DZL), University Hospital, LMU Munich, Ziemssenstr. 1, 80336 Munich, Germany; 3 Department of Medicine, Pulmonary and Critical Care Medicine, University Medical Center Giessen and Marburg, Philipps-University Marburg, Germany, Member of the German Center for Lung Research (DZL), Baldingerstrasse, 35043 Marburg, Germany; 4 Department of Internal Medicine V – Pulmonology, Allergology, Intensive Care Medicine, Saarland University Hospital, Kirrberger Straße 1, 66424 Homburg, Germany; 5 Institute of Health Economics and Health Care Management, Helmholtz Zentrum München GmbH – German Research Center for Environmental Health, Comprehensive Pneumology Center Munich (CPC-M), Member of the German Center for Lung Research, Ingolstädter Landstr. 1, 85764 Munich, Germany; 6 Hamburger Institut für Therapieforschung GmbH, Colonaden 72, 20354 Hamburg, Germany; 7 ASCONET Study Coordination Office, University of Marburg, Baldingerstraße, 35043 Marburg, Germany; 8 Department of Neurology, Klinikum Innenstadt, Ludwig Maximilian University of Munich, Ziemssenstr. 1, 80336 Munich, Germany; 9 LungenClinic Grosshansdorf, Airway Research Center North (ARCN), Member of the German Center for Lung Research (DZL), Grosshansdorf, Germany; 10 Department of General and Interventional Cardiology, University Heart Center Hamburg, Hamburg, Germany; 11 Department of Pneumology, Hannover Medical School, Carl-Neuberg-Str. 1, 30625 Hannover, Germany

**Keywords:** Peripheral neuropathy, COPD, Base excess, Ankle-brachial-index

## Abstract

**Background:**

Peripheral neuropathy is a common comorbidity in COPD. We aimed to investigate associations between alterations commonly found in COPD and peripheral neuropathy, with particular emphasize on the distinction between direct and indirect effects.

**Methods:**

We used visit 4 data of the COPD cohort COSYCONET, which included indicators of polyneuropathy (repeated tuning fork and monofilament testing), excluding patients with diabetes a/o increased HbA1c. These indicators were analysed for the association with COPD characteristics, including lung function, blood gases, 6-min walk distance (6-MWD), timed-up-and-go-test (TUG), exacerbation risk according to GOLD, C-reactive protein (CRP), and ankle-brachial index (ABI). Based on the results of conventional regression analyses adjusted for age, BMI, packyears and gender, we utilized structural equation modelling (SEM) to quantify the network of direct and indirect relationships between parameters.

**Results:**

606 patients were eligible for analysis. The indices of polyneuropathy were highly correlated with each other and related to base excess (BE), ABI and TUG. ABI was linked to neuropathy and 6-MWD, exacerbations depended on FEV_1_, 6-MWD and CRP. The associations could be summarized into a SEM comprising polyneuropathy as a latent variable (PNP) with three measured indicator variables. Importantly, PNP was directly dependent on ABI and particularly on BE. When also including patients with diabetes and/or elevated values of HbA1c (*n* = 742) the SEM remained virtually the same.

**Conclusion:**

We identified BE and ABI as major determinants of peripheral neuropathy in patients with COPD. All other associations, particularly those with lung function and physical capacity, were indirect. These findings underline the importance of alterations of the micromilieu in COPD, in particular the degree of metabolic compensation and vascular status.

## Introduction

Peripheral neuropathy (polyneuropathy) is one of the common comorbidities in COPD with a prevalence range of 5 to 100% [1–3]. In two studies, approximately one-third of COPD patients had apparent peripheral neuropathy and two-thirds showed subclinical disease [4, 5]. The question of causal connections between peripheral neuropathy and COPD is intricate as neuropathy correlates with age, just as COPD, and various other confounders. This is confirmed by mouse models of early ageing [6]. Conversely, premature ageing is considered as an important factor in the development of COPD [7]. The association between peripheral neuropathy and COPD has been explained by detrimental effects of chronic hypoxemia on peripheral nerves [8, 9], as well as systemic inflammation [10]. Among the common risk factors for COPD and neuropathy, cigarette smoke involving exposure to a variety of adverse compounds, is important [4]. In addition, diabetes, a well-known risk factor for neuropathy, seems to be more prevalent in COPD compared to the general population [11].

Beyond its pure presence as a comorbidity, peripheral neuropathy might have a functional impact in COPD, as indicated by the finding that the same degree of lung function impairment was associated with lower physical performance in patients with peripheral neuropathy compared to those without [12]. Neuropathy is also known to impair the quality of life [13], which is already lowered in COPD [14]. The differences between studies regarding the prevalence estimates of peripheral neuropathy in COPD may be partially attributed to differences between study populations, e.g. the frequency of diabetes mellitus, or differences in diagnostic or statistical procedures. Moreover, the disease COPD is characterized by alterations in many functional and clinical parameters as well as a complex network of associations between these alterations [15–18]. This raises the question, which associations with peripheral neuropathy are more or less direct and probably causative, and which are indirect and probably an expression of common underlying factors. For this purpose, the method of structural equation modelling as used previously [15, 16, 18–20] is well suited. We addressed this question using a large data set from the German COPD cohort COSYCONET (**CO**PD and **Sy**stemic **Co**nsequences - Comorbidities **Net**work) which incorporated clinically established diagnostic tools for peripheral neuropathy.

## Methods

### Study population

COSYCONET is a multi-center COPD cohort focusing on the role of comorbidities in stable COPD and initially comprising 2741 patients [21]. The present analysis was based on data of visit 4 (*n* = 1329) scheduled 3 y after inclusion, as this visit comprised the assessment of peripheral neuropathy. We only included patients with GOLD grades 1–4 with complete information on age, gender, BMI, smoking status and packyears (*n* = 1160), moreover with complete information regarding FEV_1_, FVC, RV/TLC,TLCO, mMRC and exacerbation risk (*n* = 1031) [22]. Furthermore we required complete data on ankle-brachial index (ABI), 6-MWD and TUG (*n* = 858), on CRP and leukocyte numbers (*n* = 840), on tuning fork testing, monofilament testing and neuroscore (*n* = 793), and on blood gas parameters saO_2_, CaO_2_, paO_2_, paCO_2_, BE and pH (*n* = 745). Data of three single patients were omitted based on a multivariate Mahalanobis criterion as they turned out to be completely isolated outliers (*n* = 742). Among these, we then excluded 115 patients with the diagnosis of diabetes mellitus in order to avoid interferences with peripheral neuropathy that could mask specific effects of COPD (*n* = 627) [11, 23]. For the remaining patients we required HBA1c values to be ≤6.5%. This resulted in a final dataset of *n* = 606 patients. The COSYCONET study has been approved by the ethical committees of all study centers, and all patients gave their written informed consent [21].

### Assessments

Study protocol and basic assessments of COSYCONET have been described in detail previously [21]. All patients were measured under stable conditions outside exacerbations. The diagnosis of diabetes was based on patient-reported physicians’ diagnoses and/or the presence of diabetes-specific medication [11]. Lung function data comprised forced expiratory volume in 1 s (FEV_1_), forced vital capacity (FVC), the ratio of residual volume to total lung capacity (RV/TLC), and the single-breath transfer factor for carbon monoxide (TLCO), each expressed as percent predicted [24, 25], except for RV/TLC. PaO_2_, PaCO_2_, pH, BE and SaO_2_ were determined from arterialized capillary blood of the earlobe. From these parameters the oxygen content of blood was computed, using an established formula and measured haemoglobin concentrations [18].

To quantify physical activity and the risk to fall, the 6-min walk distance (6-MWD) and the timed-up-and-go test (TUG) were used [26]. Spirometric GOLD grades were based on FEV_1_, and GOLD groups were based on exacerbation risk and symptoms according to the GOLD 2017 criteria using the mMRC [22]. The ankle-brachial index (ABI) was assessed under standardized conditions [21, 27] and evaluated as mean value from the right and left side values. Inflammation was quantified via standard assessments of CRP levels.

Peripheral neurological function was assessed via standard procedures using the Rydel-Seiffer tuning fork for the big toe basal joint (BTBJ) and end joint (EJ); the respective results were used as continuous variables. Furthermore, the monofilament testing using a 10 g filament (Tip Therm®/TwinTip), bending at a nominal force of 0.1 N, was applied at five places of the toe two times [28]. Moreover, a binary score was computed from the answers to a German questionnaire regarding peripheral neuropathy, indicating either the absence of any signs of peripheral neuropathy or the presence of at least one of them [29]. This score was only used for the description of baseline characteristics. In a subset of patients (*n* = 74), the outcome measures had been compared with measurements of nerve conduction velocity (m/s) and amplitude (μV) of nervus suralis using the NC-stat® DPNCheck in order to validate the assessments [30]. All measurements followed standardised procedures in all study centers [18, 21].

### Statistical methods

Median values and quartiles were computed for patients’ description. The two groups of patients with positive or negative neuroscore were compared with the Mann-Whitney-U-test for continuous variables; categorical data were compared using Chi-squared statistics. As most variables turned out to be dependent on age, BMI, packyears and gender within regression analyses, all subsequent computations were performed with variables adjusted for these four covariates. To detect relationships between COPD characteristics and the three quantitative parameters of peripheral polyneuropathy, multiple linear and logistic regression analyses were used. Correlation analyses were based on Spearman rank correlations. An exploratory factor analysis was used to verify the relationship between all three indices of polyneuropathy.

In combination with pathophysiological considerations, the results of these analyses were then used to construct a structural equation model (SEM) [31]. This is a combination of regression and factor analysis that is particularly suited to describe complex networks and to distinguish direct from indirect relationships. The approach has been used by us previously several times [15, 16, 18–20] and proved to be powerful. The monofilament readings were converted into a binary variable, representing values of either 10 or less than 10, which resulted in more robust results due to the skewed distribution of the monofilament readings. This variable and the two Rydel-Seiffer readings were integrated into a latent variable (construct) called “PNP”. This construction was admissible according to factor analysis and supported by the subsequent evaluations. In contrast, lung function parameters were treated separately despite their correlations with each other, as they showed quite different relationships to other variable.

For computation of the SEM, we used the generalized least squares estimation (GLE) and checked whether the results were in concordance with those obtained by the maximum likelihood or asymptotically distribution-free method; this was always true. The goodness of fit was evaluated via the comparative fit index (CFI), the root mean square error of approximation (RMSEA) and the chi-square statistics, following usual criteria [18, 19]. All analyses were performed using the software package SPSS statistics version 25 (IBM Corp., Armonk, NY, USA) and AMOS 25.0.0 (IBM Corp., Armonk, NY, USA), and the level of statistical significance was assumed at *p* < 0.05.

## Results

### Baseline characteristics

Baseline characteristics of patients are given in Table 1, including the findings regarding peripheral neuropathy. The readings for MF and EJ were significantly different between patients with and without positive scoring using the binary neuroscore, moreover there were significant differences regarding packyears, BE, CaO_2_ and 6-MWD (*p* < 0.05 each). Twenty-nine of 606 patients reported a doctor-based diagnosis of peripheral neuropathy but this subgroup was not separately analysed due to its small size.

**Table 1:** Baseline characteristics of the study cohort (*n* = 606)

Parameter	all	Negative Neuroscore	Positive Neuroscore	*p*-value
continuous	25/50/75 percentile	25/50/75 percentile	25/50/75 percentile	
Age (y)	61.0/67.0/73.0	61.0/67.0/72.0	61.0/68.0/73.0	0.311
BMI (kg/m^2^)	23.1/25.8/29.0	22.9/25.6/28.7	23.2/26.0/29.3	0.271
Packyears	20.0/40.0/61.5	16.1/37.5/57.5	23.4/42.3/66.1	**0.019**
FEV1% predicted	40.4/54.3/68.5	40.4/54.6/69.3	40.7/54.0/68.1	0.845
FVC % predicted	68.3/82.4/96.6	67.6/82.3/96.1	68.5/82.6/97.7	0.553
RV/TLC (L)	0.46/0.54/0.62	0.46/0.54/0.61	0.47/0.53/0.62	0.883
TLCO % predicted	43.4/56.6/73.6	43.3/56.7/75.7	43.4/56.4/71.6	0.614
ABI	1.12/1.21/1.29	1.13/1.22/1.30	1.11/1.20/1.28	0.098
SaO2 (%)	92.9/94.4/95.7	93.0/94.3/95.7	92.8/94.5/95.7	0.985
paO2 (mmHg)	61.6/67.1/73.4	62.0/67.5/73.0	60.9/67.0/73.5	0.704
paCO2 (mmHg)	34.7/37.0/39.6	34.6/36.9/39.0	34.8/37.2/40.0	0.109
pH	7.41/7.43/7.44	7.41/7.43/7.45	7.41/7.43/7.44	0.668
BE (mmol/l)	−0.7/0.6/1.9	−0.9/0.5/1.8	−0.5/0.9/2.0	**0.040**
CaO2 (ml/100 ml)	17.6/18.6/19.7	17.8/18.8/20.0	17.5/18.4/19.4	0.002
CRP (mg/dl)	2.0/4.0/8.0	1.8/3.6/6.7	2.0/4.0/9.6	0.136
eGFR (ml/min)	68.8/82.5/93.3	69.6/83.3/93.2	67.6/80.8/93.5	0.254
Tuning fork result BTBJ	5.4/6.5/7.5	5.5/7.0/7.5	5.0/6.0/7.5	**0.003**
Tuning fork result EJ	5.0/6.5/7.5	5.0/6.5/7.5	4.5/6.0/7.5	0.720
Monofilament result	9.5/10.0/10.0	10.0/10.0/10.0	9.0/10.0/10.0	0.310
6-MWD(m)	378/451/520	395/465/530	365/439/504	**0.010**
TUG (s)	5.40/5.42/7.60	5.1/6.4/7.4	5.7/6.5/7.9	0.122
categorical				
Gender (m/f)	362/244	201/114	161/130	**0.020**
Exacerbation risk (CD/AB)	181/425	94/221	87/204	0.529

The table shows 25th/50th and 75th percentiles of continuous parameters. For these variables the comparison between the two groups with negative or positive neuroscore was performed using the Mann-Whitney-U Test. Furthermore, categorical data for gender and exacerbation risk are given. Comparison between these groups were performed using chi-squared statistics. For abbreviations see text

Significant differences are marked in bold. The level of statistical significance was set at *p*<0.05

### Relationship between parameters

BTBJ, EJ and MF were correlated with each other (*p* < 0.001 each). The relationship between the three parameters was confirmed by factor analysis showing that all variables belonged to one component (explained variance 72.6%); this was also true after adjustment for age, BMI, packyears and gender (explained variance 71.4%); this justified that in the construction of the SEM these three variables were assigned to a latent variable.

An analogous correlation analysis was performed to understand the relationship between functional parameters, always using values adjusted for age, BMI, packyears and gender. There were significant associations (*p* < 0.01 each) between FEV_1_, FVC, RV/TLC, TLCO, 6-MWD and ABI. Furthermore, FEV_1_, RV/TLC and TLCO were correlated with BE, paO_2_ and saO_2_ (*p* < 0.001 each). 6-MWD correlated with all blood gas parameters except pH (*p* < 0.05), whereas ABI was only related to 6-MWD (*p* < 0.001) in these analyses.

In the next step the relationships to the three measures of polyneuropathy, as dependent variables, were determined by stepwise linear regression analyses, again using values adjusted for age, BMI and gender. We additionally included blood gas parameters and CRP as predictors. BTBJ was linked to ABI and BE, EJ to TUG and BE, and the monofilament reading to TUG (*p* < 0.05 each). Conversely, BE was dependent on BTBJ, oxygen saturation and RV/TLC (*p* < 0.05 each); in these analyses, paCO_2_ and pH were omitted due to their trivial correlation with BE. 6-MWD depended on FEV_1_, TLCO, ABI, TUG, exacerbation risk and CRP (*p* < 0.01 each); FVC was omitted in this and further analyses due to its high collinearity with FEV_1_. The ABI was dependent on EJ and 6-MWD (*p* < 0.05), with a tendency towards an additional dependence on TLCO (*p* = 0.052). Exacerbation risk was dependent on FEV_1_, 6-MWD and CRP (*p* < 0.05 each).

### Structural equation model

**Fig. 1:**
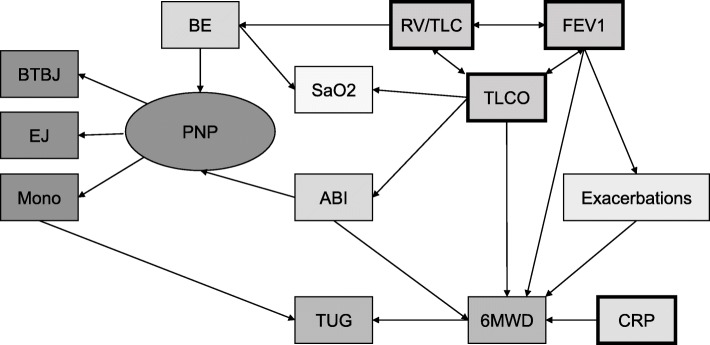
Structural equation model (SEM). For regression and correlation coefficients see Table 2. Unidirectional arrows indicate a relationship in terms of a linear regression, and double-headed arrows a correlation between variables. All rectangles represent observed variables, whereby the error terms for dependent variables (those, to which at least one arrow points) have been omitted for the sake of clarity. The oval represents a latent variable (construct PNP) comprising the three peripheral neuropathy measures as indicator variables. The structure of relationships illustrates that BE and ABI had effects on the total construct PNP, whereas the lung function parameters showed different relationships to other variables and therefore could not be summarized into a construct

On the basis of these findings, we constructed a structural equation model which is shown in Fig. 1. The respective regression coefficients are shown in Table 2. In building the model, we followed the same criteria as in our previous work [15, 16, 18–20] and aimed at obtaining a model which was both statistically robust and physiologically meaningful. The particular relationships and their direction (as indicated by the arrowheads) were chosen based on the results of the correlation or regression analyses and, in case of ambiguities, supplemented by pathophysiological considerations. All links that turned out to be non-significant were omitted.

**Table 2:** Results of the structural equation modelRegression

			Estimate	S.E.	C.R.	Standardized.	*P*
ABI	←	TLCO	.001	.000	4.600	.185	*p* < 0.001
BE	←	RV/TLC	5.829	.800	7.284	.291	*p* < 0.001
PNP	←	BE	.082	.035	2.372	.100	.020
PNP	←	ABI	1.139	.535	2.128	.091	.033
Exacerbations	←	FEV_1_	−.005	.001	−5.346	−.216	*p* < 0.001
6-MWD	←	ABI	97.583	24.735	3.945	.130	*p* < 0.001
6-MWD	←	FEV_1_	1.713	.229	7.493	.303	*p* < 0.001
6-MWD	←	TLCO	1.442	.194	7.440	.297	*p* < 0.001
6-MWD	←	Exacerbations	−26.189	7.665	−3.417	−.115	*p* < 0.001
Monofilament	←	PNP	−.079	.010	−7.562	−.322	*p* < 0.001
6-MWD	←	CRP	−25.565	7.507	−3.406	−.113	*p* < 0.001
End joint (EJ)	←	PNP	1.000			.904	
Basal joint (BTBJ)	←	PNP	1.023	.076	13.413	.933	*p* < 0.001
SaO_2_	←	TLCO	.027	.004	6.564	.254	*p* < 0.001
SaO_2_	←	BE	−.234	.042	−5.501	−.214	*p* < 0.001
TUG	←	6-MWD	−.010	.001	16.231	−.562	*p* < 0.001
TUG	←	Monofilament	.359	.152	.018	.082	*p* < 0.001
Covariances			Estimate	S.E.	C.R.	Standardized	*P*
FEV_1_	↔	TLCO	214.898	18.125	11.857	0.558	*p* < 0.001
TLCO	↔	RV/TLC	−.923	.099	−9.294	−0.416	*p* < 0.001
FEV_1_		RV/TLC	−1.398	.098	−14.216	−0.734	*p* < 0.001

The upper panel refers to the directed arrows (regression terms) depicted in Fig. 1, whereby the left part lists the arrows shown in this figure. The right part shows the results of the corresponding statistical tests. The first column of the right part shows the non-standardized estimate of the respective regression coefficient, the second column the standard error (S.E.) of this coefficient, the third column the ratio of these two values (critical ratio, C.R.) which is used for significance testing. The fourth column shows the standardized estimates of the regression coefficients shown in the first column. The last column shows the significance level based on the generalized least squares (GLS) procedure of AMOS. In an analogous manner the lower panel shows the covariances (bidirectional arrows in Fig. 1) between lung function parameters, as well as the respective standard errors, critical ratios, correlation coefficients and significance levels. In the SEM, CRP values were logarithmically transformed (log10) after addition of 0.05, in order to account for the skewness of data and obtain a distribution closer to normal. For abbreviations of symbols see text

The three measures of polyneuropathy were implemented as indicators of a latent variable called PNP. Lung function parameters were kept separately as they showed markedly different relationships to the other parameters, in accordance with physiological expectations. To account for their mutual correlations, these were explicitly included into the model (indicated by double-headed arrows). Regarding their effects on other parameters, RV/TLC had an effect on BE, FEV_1_ on exacerbation risk and 6-MWD, and TLCO on 6-MWD, ABI and oxygen saturation. The ABI was dependent on TLCO, while it had effects on 6-MWD and PNP. 6-MWD was dependent on exacerbations, FEV_1_, TLCO and ABI, while it influenced TUG. Exacerbation risk only depended on FEV_1_ but affected 6-MWD, which was also dependent on CRP (which was logarithmically transformed due to its skewed distribution, see legend to Table 2). PNP was dependent on BE and ABI, whereby there was an additional link from the monofilament readings to TUG. In addition, TUG was dependent on 6-MWD. Oxygen saturation was dependent on TLCO and BE; compared to other parameters of oxygen supply (saO_2_, paO_2_, CaO_2_) oxygen saturation turned out to be the best correlated and most informative parameter, thus the other parameters were omitted.

The final model fitted with an CFI of 0.991, an RMSEA of 0.013 (90%CI 0.000; 0.029), and chi-square value of 63.553, with 58 degrees of freedom (*p* = 0.287). When the directions of single arrows were inverted, in all cases the reversion resulted in a reduction or loss of statistical significance, thereby underlining the validity of the model.

### Sensitivity analysis

The results described above were obtained for patients without the diagnosis of diabetes and/or elevated (> 6.5%) values of HbA1c. When the complete analysis was repeated including patients with diabetes and/or elevated HbA1c (*n* = 742), virtually the same results were obtained, and all dependences within the structural equation model were still significant. This model fitted with a CFI of 0.980, an RMSEA of 0.019 (90%CI 0.000; 0.031), and chi-square value of 73.867, with 58 degrees of freedom (*p* = 0.078). Conversely, when 13 of 606 patients who had reported a history of alcohol abuse were omitted, all links were still significant, especially that from BE to PNP (*p* = 0.024), while the link from ABI to PNP was at the border of significance (*p* = 0.072). We performed two different sensitivity analyses to account for possible associations between metabolic changes linked to renal impairment especially with PNP and BE. First, we additionally introduced kidney function in terms of glomerular filtration rate (GFR) as computed according to Levey et al. [32], all associations shown in Fig. 1 remained significant, while GFR showed associations with both, PNP and BE (*p* = 0.015 and 0.004, respectively), and a satisfying overall fit (CFI = 0.963, *n* = 604). International guidelines define chronic kidney disease as (a) GFR < 60 ml/min per 1.73 m^2^ or (b) markers of kidney damage, or both, of at least 3 months duration [33]. In a second step we thus repeated the complete analysis (see Fig. 1) excluding all patients with GFR < 60 ml/min which resulted in *n* = 529 patients. All dependences within the structural equation model remained significant. This model fitted with a CFI of 0.991, an RMSEA of 0.013 (90% CI 0.000; 0.030), and chi-square value of 63.042, with 57 degrees of freedom (*p* = 0.303). Especially the link from BE to PNP remained significant (*p* = 0.026), again confirming the robustness of the SEM.

## Discussion

Peripheral neuropathy is a common disorder particularly in the elderly, with multiple risk factors and associations between them. This renders it difficult to identify relevant factors, particularly in diseases not implicating an overwhelming increased risk such as diabetes. COPD probably belongs to these diseases, and this might explain the heterogeneity of associations and prevalence estimates in the literature. In the present analysis, we aimed to identify factors associated with COPD that are related to peripheral neuropathy. There are multivariate, advanced statistical techniques that allow to disentangle multiple relationships even within cross-sectional data and at least to get clues on direct and indirect influencing factors. Especially if applied to a population of COPD patients without the major risk factor diabetes, that would help to identify genuine effects of COPD and to separate them from effects not specific of COPD, for example arising from age or diabetes. To obtain a picture as clean as possible, we always adjusted for common risk factors, in particular age, and omitted patients with diabetes or elevated values of HbA1c.

The assessment of neuropathy was based on three functional measures, which could be summarized into a combined variable called PNP. One of the indicators of polyneuropathy was directly linked to a functional measure indicating the patient’s risk of falling; this association appeared plausible, although not involving a COPD-specific measure. There were, however, two COPD-related characteristics that were directly linked to PNP in stable COPD: an increased base excess and a decreased ABI. All other measures of COPD morbidity, in particular those of airway obstruction, lung hyperinflation, gas exchange capacity, oxygen saturation, physical capacity, exacerbation risk and CRP levels, were only indirectly linked to PNP. This suggests that some correlations of peripheral neuropathy with COPD indices reported in the literature were probably also indirect. Our findings suggest that especially base excess and ABI are markers of those chronically distorted systemic conditions in COPD, which have an impact on the development and degree of peripheral neuropathy, probably by affecting the micromilieu of nerves.

Several animal studies found that acute and chronic ischaemia can lead to peripheral nerve lesions [34–37], in accordance with clinical observations indicating a higher risk for peripheral neuropathy in COPD patients with hypoxemia [38]. These findings are in accordance with occurrence of subclinical peripheral neuropathy in relation to hypoxemia in COPD [39]. Vascular changes are considered as a major determinant underlying peripheral neuropathy [40–42]. Especially in human diabetic neuropathy, impaired nerve blood flow, epineurial arterio-venous shunting and a reduction in sural nerve oxygen tension are thought to play a role [40]. The vascular involvement shown in diabetes is in line with our result obtained in patients without diabetes, showing a link between the ankle-brachial index and the degree of polyneuropathy. The ankle-brachial index is considered as marker of macrovascular changes but is also indicative of microvascular alterations [43, 44], this might be one of the reasons why we observed an association with diffusing capacity. Oxygen saturation, as a measure of ischemia, was not directly related to peripheral neuropathy but indirectly via base excess. Elevated base excess is a sign of long-term respiratory impairment and was associated with an elevated degree of polyneuropathy. It indicates the need for long-term metabolic compensation in COPD, including lung, kidney function and acid-base balance. The central role of BE in stable COPD has been illustrated regarding renal impairment and exacerbations [18]. This study also showed its association with impaired lung function. This was confirmed in the present analysis comprising a different subpopulation and study visit of COSYCONET patients. Probably an elevated BE reflects the compensation of intermittent but chronic nocturnal hypoxemia, as demonstrated for patients with obesity hypoventilation syndrome [45]. In this respect, in stable COPD BE seems to be a marker of long-term distortions of the internal environment that are important for various comorbidities including peripheral neuropathy. Possibly, in this regard BE is superior to spirometric lung function that has not been found to be associated with polyneuropathy if taken as only characteristic of COPD [46].

Metabolic factors, i.e. the internal environment, are known to be relevant in diabetes, interacting synergistically with vascular factors [40, 41, 47]. Their combination probably causes the high frequency of peripheral neuropathy in diabetes, by altering the microenvironment of nerves. To identify genuine COPD-related factors as clearly as possible, we excluded patients with a clinical history of diabetes, based on patients’ reports of physicians’ diagnoses and/or the intake of diabetes-specific medication [23]. Moreover, we excluded a number of undiagnosed patients with elevated values of HbA1c. When including patients with diabetes and/or elevated values of HbA1c the results remained the same. We therefore consider it likely, that the links between COPD characteristics and peripheral neuropathy, which we found, represent causal relations that are not due to confounding factors such as diabetes [11]. When excluding patients with a history of alcohol abuse as risk factor for peripheral neuropathy, the link between BE and PNP remained significant, while the association with ABI became weaker. This underlines the role of BE as a COPD-related determinant. An additional factor related to BE and PNP was kidney function in terms of GFR [32] but this did not eliminate the role of BE per se for PNP, either when including eGFR into the analysis or when excluding patients with eGFR< 60 ml. The observed association between GFR and BE was in line with previous findings focussing on GFR [18]. As kidney function is also well known to be associated with peripheral neuropathy [48], we decided to omit GFR from the model presented in the present analysis.

Previous studies have reported an association between physical capacity and peripheral neuropathy in COPD [42]. Although we could not identify a direct link between these two measures, there were indirect links, particularly mediated via vascular function. Abnormalities in blood vessel walls play a role for both peripheral artery disease [49] and peripheral neuropathy [50], whereas peripheral artery disease is known to be related to physical capacity [27]. As measure of the risk of falling that is well established in geriatric research, we included the timed-up-and-go test. The values obtained were dependent on physical capacity, which seems fully plausible, but interestingly they were also related to the monofilament result. This also seems plausible, as a reduced pallesthesia is likely to be associated with unsteadiness in walking which is reflected by the timed-up-and-go test. The finding that the monofilament testing was a relevant measure, may reflect the suitability of this test for higher degrees of severity compared to the tuning fork. Other determinants of TUG as identified in geriatric populations did not seem to play a major role, in particular as we adjusted for age.

CRP concentrations, as a systemic marker of inflammation, were linked to physical capacity but not directly to peripheral neuropathy. The absence of a direct relationship between peripheral neuropathy and inflammation is in line with a previous study that analysed inflammatory biomarkers including CRP for their association with pulmonary function and arterial stiffness [51], which we found to be directly and indirectly associated with neuropathy. This study found no significant associations after adjustments for age, sex, height, ethnicity, BMI, smoking status and history, suggesting that systemic inflammation plays a secondary role for neuropathy [51]; this may be different for acute nerve injury [52].

A common risk factor for COPD, vascular diseases and peripheral neuropathy is age [38]. We adjusted for age, in addition for gender, packyears and BMI, thus the relationships identified by us were not due to age as a common factor. Despite this, age might play a role in terms of premature aging. Age-related impairments of elastic fibers of the arterial wall may induce peripheral artery disease [53], and the corresponding alterations of the microvascular environment of peripheral nerves may promote neuropathy. At the same time, these impairments may alter the elastic recoil of the lung, with potential impact on the development of emphysema [54], while emphysema promotes hypoxic conditions. This interplay could become important in COPD in the presence of pre-mature aging [7], thereby explaining why we observed relationships despite of the adjustment for age.

The validity of the measurements regarding peripheral neuropathy had been checked in a subset of patients (*n* = 74) from the initial COSYCONET visits using direct measurement of nerve conduction velocity [30]; indeed, the results of the tuning fork and monofilament testing correlated with the velocity measurements. We therefore assume that the latent variable PNP comprising the results of the tuning fork and monofilament testing adequately described the presence and degree of peripheral neuropathy.

### Limitations

Naturally, the present cross-sectional analysis does not allow to infer causal relationships, although the directionality of the SEM may be suggestive of causal associations. The tuning fork and monofilament tests are well established in clinical practice but may not be the most sensitive measures, especially compared to nerve conduction velocity. Overall, the alterations identified by us were largely subclinical and the associations were weak as indicated by the standardized regression coefficients. Despite this, the robustness of the findings when including diabetes patients, as well as their consistency with known data from patients with and without COPD, suggests that these associations were valid. It is unlikely, that interference with neuropathy-inducing medication was relevant in our study population, especially when considering the relatively low frequency of potentially problematic medications in COSYCONET [55]. Moreover, all variables were adjusted for the shared risk factors age, BMI, packyears and gender, rendering it unlikely that these common factors were underlying the observed associations. The diagnostic instruments used by us to quantify peripheral neuropathy mainly detected the presence of sensitive disorders. Despite this, we probably not completely missed motorical disabilities associated with neuropathy as we included the 6-MWD and the TUG as measures of physical function and the TUG result was directly linked to the monofilament result.

## Conclusion

Using data from the COSYCONET cohort, we analysed peripheral neuropathy in stable COPD patients without diabetes, with the aim to identify those COPD characteristics that were the primary determinants of neuropathy. It turned out that base excess and ankle-brachial index, which are often found to be impaired in COPD patients, were the most relevant factors related to peripheral neuropathy. Other associations, especially those with lung function, oxygen saturation and exercise capacity were only indirect and mediated by these determinants. Circulatory disorders and the long-term metabolic compensation of respiratory acidosis could therefore contribute to the occurrence of polyneuropathy in COPD. These findings underline the role of the internal environment, including the microenvironment of peripheral nerves, for the development of comorbidities in COPD. A potential clinical conclusion could be that patients with marked abnormalities in base excess and vascular function should be screened for peripheral neuropathy.

## Data Availability

The basic data are part of the German COPD cohort COSYCONET (www.asconet.net/) and available upon request. There is a detailed procedure for this on the website of this network. Specifically, the data can be obtained by submission of a proposal which is evaluated by the steering committee. All results to which the manuscript refers to are documented by the appropriate in the text, figures or tables.
